# Birth Rate as a Determinant of Dementia Incidence: A Comprehensive Global Analysis

**DOI:** 10.1177/15333175241287677

**Published:** 2025-01-29

**Authors:** Wenpeng You

**Affiliations:** 1 Adelaide Medical School, 1066 The University of Adelaide, Adelaide, SA, Australia; 2 School of Nursing and Midwifery, Western Sydney University, Sydney, NSW, Australia; 3 Adelaide Nursing School, 1066 The University of Adelaide, Adelaide, SA, Australia

**Keywords:** birth rate, dementia incidence rate, predictive influence, family size, global health

## Abstract

**Background:**

The role of parity in predicting dementia risk in women is debated. This study examines how birth rate affects global dementia incidence.

**Methods:**

Country-specific data on birth rate and dementia incidence rate were analyzed using bivariate analysis, partial correlation, and multiple linear regression. Confounding factors such as aging, affluence, genetic predisposition (I_bs_), and urbanization were considered.

**Results:**

Pearson’s r and nonparametric analyzes showed a significant inverse correlation between birth rate and dementia incidence. This relationship remained significant after controlling for aging, affluence, I_bs_, and urbanization. Multiple linear regression identified birth rate as a significant predictor of dementia incidence, and as the strongest predictor. Affluence and urbanization were not significant predictors. The correlation was stronger in developing countries.

**Conclusions:**

Lower birth rate is an independent risk factor for dementia, particularly in developed countries. These findings highlight the importance of considering birth rate in dementia studies.

## Introduction

Dementia is a condition that affects memory, language, and cognition. It has a global impact, with over 55 million people affected and nearly 10 million new cases diagnosed each year.^1^ Historically, dementia has been referenced in medical texts dating back to the second century, but it was not until the early 20^th^ century that Alois Alzheimer identified the condition as a distinct neurodegenerative disease, paving the way for modern research into its causes and treatment. Despite advancements in dementia research, there is still a significant lack of awareness about the condition. This gap in understanding contributes to stigmatization and creates barriers to care.^1^ This often results in the denial of basic rights for individuals with dementia.^1^ The World Health Organization (WHO) has highlighted the substantial physical, psychological, social, and economic impacts of dementia and recognizes it as a public health priority.^1^

The COVID-19 pandemic has further complicated dementia care.^2-4^ Studies have shown that dementia patients were more vulnerable to severe outcomes from COVID-19, experiencing higher mortality rates and worsened cognitive symptoms due to social isolation and disruption in care.^3^ Emerging research also suggests that COVID-19 infection may increase the risk of developing dementia due to neuroinflammatory responses. For instance, one study found that survivors of COVID-19 have an elevated risk of cognitive decline, particularly those with preexisting conditions.^3,4^ Another study highlighted that the pandemic disproportionately affected individuals with dementia, worsening their mental health and accelerating cognitive deterioration.^3,5^ These findings underscore the intersection of global health crises and dementia care, further emphasizing the urgency for public health interventions.

Aging and genetics are well-known risk factors for dementia.^6^ The role of genetic predisposition in dementia is still under investigation. However, evidence suggests that genetic factors may accumulate at the population level, potentially increasing dementia incidence.^7^ The global shift towards an aging population, driven by improved healthcare, has increased the proportion of older individuals. As a result, dementia primarily affects this growing age group.^1^ Furthermore, there is a growing awareness of cases of early-onset dementia occurring before the age of 65.^8^

Parity refers to the number of pregnancies a woman has carried to a viable gestational age. Birth rate measures the number of live births per 1000 people annually and indicates female fertility at the population level. Both of these metrics are related to a woman’s childbearing and can indicate family size.^9-13^ These indicators have been widely associated with the risk of developing non-communicable diseases, such as cancer^14^ and dementia.^15^

Research on the impact of parity on dementia risk at an individual level is limited and presents mixed results.^16^ Some studies suggest that higher parity is associated with an increased risk of dementia.^17^ In contrast, other research indicates a lower risk for women with no children^18^ or those with three or more childbirths compared to women who have given birth once.^19^ These inconsistent findings were also observed in a study that examined 11 populations on three continents.^16^

These studies may have inherent biases. Firstly, they only associated a mother’s dementia risk with the number of children she has, without considering the impact on the health of all family members. Larger family size, known as grand parity, has been shown to have protective effects against the development of non-communicable diseases, including dementia.^15^ Secondly, psychological factors, such as stress from adverse life events, were suggested to increase dementia risk.^20^ Dementia patients tend to recall negative life events more easily, which contributes to this risk.^21^ Positive psychological experiences, such as pregnancy and childbirth, may enhance life satisfaction. These experiences can increase oxytocin production, which may help inhibit the onset of dementia^22,23^. The combined therapeutic effects of oxytocin and the psychosocial support from larger families may reduce dementia risk in both males and females.^15,24^ However, these factors were not considered in the studies.

Given the conflicting and circumstantial findings of previous studies, this research aims to assess the predictive influence of birth rates on total dementia incidence rates from both global and regional perspectives. This study will use empirical, macro-level data from international organizations. It will incorporate major risk factors, such as aging, economic affluence, genetic predisposition, and urbanization, to analyze the relationship between birth rate and dementia incidence rates. The goal is to clarify how birth rate affects dementia risk. This analysis will consider demographic trends and their implications for public health planning and policymaking.

## Materials and Methods

### Data Sources

A comprehensive list of dementia incidence rates in 204 countries was obtained from the Institute for Health Metrics and Evaluation of the University of Washington (IHME). To analyze this data, five additional variables were extracted and matched with the country list using country-specific data published by the United Nations and its agencies. In the terminology of international organizations, the terms “location,” “population,” and “country” are interchangeable and refer to a single data reporting unit.

In this study, the dependent variable is the dementia incidence rate (DIR), sourced from the Institute for Health Metrics and Evaluation of the University of Washington.^25^ The DIR is expressed as the number of newly diagnosed dementia cases per 100,000 people in 2019.

The independent variable, birth rate, was downloaded from the World Bank data repository.^26^ Birth rate indicates the number of live births per 1000 population estimated at midyear of 1999. It is used to index fertility in the study, specifically looking at delayed presentation of dementia. The terms “birth rate” and “fertility” are used interchangeably in this paper.

Based on previous studies, four potential confounding variables were included to analyze the independent role of birth rate in predicting DIR:

1. Economic affluence, indexed with per capita GDP purchasing power rate (GDP PPP in 2018 international $), was chosen and downloaded from the World Bank data repository^26^ because it is associated with dementia risk and diagnose.^27^ This variable takes into account the relative cost of local goods, services, and inflation rates of the country.
2. Life expectancy at birth, which reflects the aging process at the population level, was downloaded from the World Bank data repository.^28^ Although dementia can occur at any stage of life, it predominantly affects older individuals. Therefore, life expectancy at birth in 2018 is used to index the aging process.
3. The Biological State Index (I_bs_), which measures the magnitude of dementia gene accumulation in a population, was downloaded from a previous publication in 2018.^29^ It is postulated that reduced natural selection (measured by I_bs_) may have allowed the accumulation of deleterious genes related to non-communicable diseases like dementia.^15^
4. Urbanization data, represented by the country-specific percentage of the population living in urban areas in 2018,^30^ was sourced from the World Bank data repository. Urbanization is a significant predictor of dementia because it reflects major demographic shifts and lifestyle changes. It also indicates the level of healthcare access within a country.^31,32^

## Data Selection

A comprehensive list of “countries” was generated from IHME, consisting of countries with dementia incidence rates.^33^ The list comprises a total of 204 “countries”. Following this, the other variables were individually matched with the list. Birth rate, aging, economic affluence, I_bs_, and urbanization data were collected for all countries where available.

Each country was treated as a separate study subject during the data analysis. It is important to note that not all countries had information for all variables. In this study, the term “country” does not refer to a sovereign nation, but rather to a reporting unit within various international organizations.

### Data Analysis

To assess the predictive influence of birth rate on DIR at the population level, the analysis was conducted in five steps.^34,35^

1. Scatter plots were generated in Microsoft Excel^®^ using the original data to visually analyze the correlation between birth rate and DIR. These plots also helped assess data quality by showing the distribution and relationships within the dataset.
2. Bivariate correlations (Pearson’s r and nonparametric Spearman’s rho) were performed to evaluate the direction and strength of the correlations between all variables. These correlations also considered the potential effects of non-normal distributions on the strength of moment-product correlations.
3. Partial correlation analysis using Pearson’s moment-product approach was conducted. Each of the six variables (aging, birth rate, economic affluence, I_bs_, DIR, and urbanization) was alternated as the independent predictor while the other five variables were included as potential confounding factors. Fisher’s r-to-z transformation was used to assess the significance level of differences between pairs of correlation coefficients.
4. Standard multiple linear regression (enter) was used to describe the correlations between the dependent variable (birth rate) and the predicting variables. To explore if low birth rate can partially explain the correlations between aging, economic affluence, I_bs_, urbanization, and DIR, enter multiple linear regression was performed with two models: (1) incorporating birth rate as a predicting variable and (2) excluding birth rate as a predicting variable. Additionally, standard multiple linear regression (stepwise) was used to select the predicting variable(s) with the greatest influence on DIR in two versions: (1) incorporating birth rate and (2) excluding birth rate.
5. To ensure a comprehensive understanding of the correlation between birth rate and DIR across different global contexts, the study conducted analyzes with grouped populations. This grouping allowed for a comparative analysis of the correlation strengths between birth rate and DIR across various country classifications. Countries were categorized based on various criteria, including World Bank income classifications (low- and middle-income countries and high-income countries), United Nations and WHO regional classifications, and other groups determined by geography, culture, development role, or socioeconomic status. Examples of specific groupings included the Asia Cooperation Dialogue, Asia-Pacific Economic Cooperation, the Arab World, the European Economic Area, countries where English is the official language, Latin America, Latin America and the Caribbean, the Organization for Economic Co-operation and Development, the Southern African Development Community, and the Shanghai Cooperation Organization. Each of these groupings was chosen to represent distinct characteristics that could impact health outcomes, including DIR. The DIR data for each grouping was obtained from the respective organizations’ official websites. This allowed for a detailed analysis of how geographical and socioeconomic factors influence dementia incidence globally.

The World Health Organization reports that over 55 million people worldwide have dementia. More than 60% of these individuals live in low- and middle-income countries. To analyze this, a Fisher r-to-z transformation was performed. This transformation compared the role of birth rate in predicting dementia across different income levels. It also examined differences between United Nations (UN)-developing and UN-developed countries.

Various statistical techniques were used to analyze the data, including Pearson’s correlation and Spearman’s rho correlation. Additionally, partial correlation and multiple linear regression (both enter and stepwise methods) were employed. The Statistical Package for the Social Sciences (SPSS) version 29 was utilized for these computations. Statistical significance was reported at a *P*-value of less than 0.05, with additional levels of significance reported for *P* < 0.01 and *P* < 0.001. The regression analysis criteria were set at a probability of F to enter less than or equal to 0.05 and to remove greater than or equal to 0.10. Scatter plots were created in Excel^®^ 2016 using the raw data.

## Results

**Figure 1.**
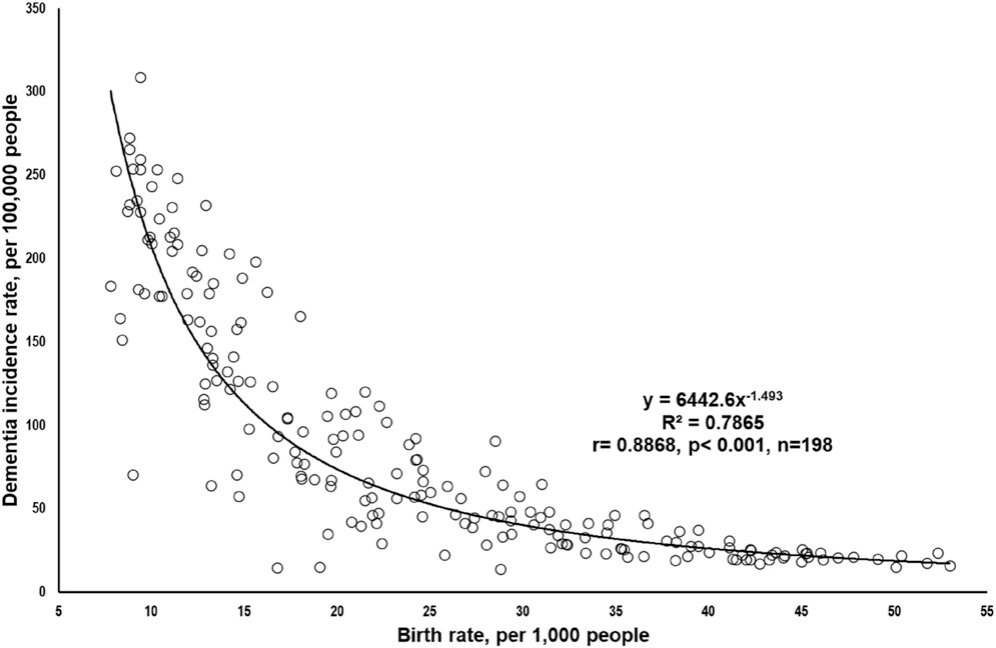
The statistical relationship between birth rate and dementia incidence rate. Data source & definition: Birth Rate: Crude birth rate, indicating the number of live births per 1000 population during the year, estimated at midyear. Source: The World Bank; Dementia Incidence Rate: The number of new cases per 100,000 people diagnosed in 2019. Source: Institute for Health Metrics and Evaluation, University of Washington.

The scatterplots showed a polynomial relationship between birth rate and DIR. There was a strong negative correlation, as indicated by an *R*^2^ value of 0.7865 (*P* < 0.001, n = 198, Figure 1).

The scatterplots confirmed the strong relationship between birth rate and DIR. This relationship was further confirmed through nonparametric and Pearson r analyzes using log-transformed data.

On a global scale, the birth rate showed a significant negative correlation with DIR. This was evident in both the Pearson analysis (r = −0.797, *P* < 0.001) and the non-parametric analysis (rho = −0.910, *P* < 0.001) as shown in Table 1.

**Table 1.** Bivariate Correlation Coefficients Between all Variables (Pearson r Correlation Coefficient Above the Diagonal and the Nonparametric Rho Correlation Coefficient Below the Diagonal).

	Birth rate aging	Dementia incidence	I_bs_	Economic affluence	Urbanization	Aging
Birth rate	1	−0.797	−0.844	−0.653	−0.558	−0.851
Dementia incidence	−0.910	1	0.590	0.582	0.483	0.717
I_bs_	−0.878	0.833	1	0.567	0.523	0.876
Economic affluence	−0.840	0.759	0.895	1	0.649	0.733
Urbanization	−0.541	0.510	0.630	0.720	1	0.604
Aging	−0.833	0.810	0.930	0.880	0.640	1

Pearson r (above diagonal) and nonparametric (below diagonal) correlations were reported. Significance levels:

***P < 0.001. Number of country range: 181-198.

Data source & definition: Birth Rate: Crude birth rate, indicating the number of live births per 1000 population during the year, estimated at midyear. Source: The World Bank; Dementia Incidence Rate: The number of new cases per 100,000 people diagnosed in 2019. Source: Institute for Health Metrics and Evaluation, University of Washington; Economic Affluence: Measured by per capita GDP (PPP), which is the per capita purchasing power parity value of all final goods and services produced within a territory in a given year (2018). Source: The World Bank; Biological State Index (I_bs_): Represents the level of genetic accumulation related to dementia due to reduced natural selection. Source: You & Henneberg, 2018; Life Expectancy at Birth (Aging): Source: The World Bank; Urbanization: Measured by the percentage of the population living in urban areas in 2018. Source: The World Bank.

Additionally, it was found that aging, affluence, I_bs_, and urbanization had moderate to strong correlations with DIR. These correlations were significant in both the Pearson and non-parametric analyzes as shown in Table 1.

The relationship between DIR and each potential confounding variable (aging, birth rate, affluence, I_bs_, and urbanization) was examined by conducting a partial correlation analysis. In this analysis, the other five variables were held constant statistically. The results revealed a strong and significant correlation between DIR and birth rate (r = −0.674, *P* < 0.001) regardless of the other variables (Table 2). Aging and I_bs_ also showed significant correlations with DIR, although these correlations were weak to moderate (r = −0.429 and 0.315, *P* < 0.001 respectively). On the other hand, economic affluence and urbanization had minimal correlations with DIR, indicating that they did not independently correlate with DIR.

**Table 2.** The Comparative Partial Correlation Coefficients for the Dementia Incidence Rate and the Predicting Variables. The Coefficients Are Examined Across Different Combinations of Controlled Variables.

Variables	Table 2-1: Birth rate, economic affluence, I_bs_, Aging and urbanization were alternated as the predicting variable for calculating its independent relationship with dementia incidence while the other three variables are statistically kept constant														
	Dementia incidence			Dementia incidence			Dementia incidence			Dementia incidence			Dementia incidence		
	r	p	df	r	p	df	r	p	df	r	p	df	r	p	df
Birth rate	−0.674	<0.001	174	-	-	-	-	-	-	-	-	-	-	-	-
Economic affluence	-	-	-	−0.092	0.224	174	-	-	-	-	-	-	-	-	-
I_bs_	-	-	-	-	-	-	−0.429	<0.001	174	-	-	-	-	-	-
Aging	-	-	-	-	-	-	-	-	-	0.315	<0.001	174	-	-	-
Urbanization	-	-	-	-	-	-	-	-	-	-	-	-	0.055	0.465	174

Variables	Table 2-2: Birth rate, economic affluence, I_bs_, aging and urbanization were alternated as the individual potential confounder for exploring the partial correlations between dementia incidence and the other four independent variables														
	Dementia incidence			Dementia incidence			Dementia incidence			Dementia incidence			Dementia incidence		
	r	p	df	r	p	df	r	p	df	r	p	df	r	p	df
Birth rate	-	-	-	−0.698	<0.001	177	−0.713	<0.001	177	−0.545	<0.001	177	−0.747	<0.001	177
Economic affluence	0.096	0.200	177	-	-	-	0.353	<0.001	177	0.100	0.184	177	0.405	<0.001	177
I_bs_	0.087	0.244	177	0.525	<0.001	177	-	-	-	−0.100	0.184	177	0.467	<0.001	177
Aging	−0.298	<0.001	177	0.398	<0.001	177	0.496	<0.001	177	-	-	-	0.614	<0.001	177
Urbanization	0.063	0.399	177	0.156	<0.050	177	0.224	<0.010	177	0.070	0.350	177	-	-	-

Data source & definition: Birth Rate: Crude birth rate, indicating the number of live births per 1000 population during the year, estimated at midyear. Source: The World Bank; Dementia Incidence Rate: The number of new cases per 100,000 people diagnosed in 2019. Source: Institute for Health Metrics and Evaluation, University of Washington; Economic Affluence: Measured by per capita GDP (PPP), which is the per capita purchasing power parity value of all final goods and services produced within a territory in a given year (2018). Source: The World Bank; Biological State Index (I_bs_): Represents the level of genetic accumulation related to dementia due to reduced natural selection. Source: You & Henneberg, 2018; Life Expectancy at Birth (Aging): Source: The World Bank; Urbanization: Measured by the percentage of the population living in urban areas in 2018. Source: The World Bank.

Standard multiple linear regression analysis was conducted to predict DIR, considering aging, birth rate, economic affluence, I_bs_, and urbanization as the predicting variables.

When birth rate was excluded as a predicting variable, only aging showed a significant correlation with DIR (Beta = 0.750, *P* < 0.001). However, when birth rate was included as an independent predictor, birth rate, I_bs_, and aging all showed significant correlations with DIR (Beta = - 0.955, - 0.613, and 0.478 respectively, *P* < 0.001). None of the other five predictors showed a strong and significant correlation with DIR (Table 3).

**Table 3.** Factors That Independently Predict the Incidence Rate of Dementia, as Determined by Multiple Linear Regression Modelling.

3-1: Enter	Dependent variable: Dementia incidence rate; All Countries, n = 204		Dependent variable: Dementia incidence rate all Countries, n = 204		
Predictor	Beta, standardized	Sig	Predictor	Beta, standardized	Sig
Birth rate	Not applicable		Birth rate	−0.955	<0.001
Economic affluence	0.064	0.457	Economic affluence	−0.082	0.224
I_bs_	−0.124	0.279	I_bs_	−0.613	<0.001
Aging	0.750	<0.001	Aging	0.478	<0.001
Urbanization	0.041	0.563	Urbanization	0.039	0.465

3-2: Stepwise	Dependent variable: Dementia incidence rate; all countries, n = 204		Dependent variable: Dementia incidence rate All countries, n = 204		
Rank	Predictor	Adjust *R*^2^	Rank	Predictor	Adjust *R*^2^
1	Birth rate	Not incorporated	1	Birth rate	0.648
	Aging	0.504	2	Ibs	0.678
	Economic affluence	Not significant	3	Aging	0.711
	I_bs_	Not significant		Economic affluence	
	Urbanization	Not significant		Urbanization	

Data source & definition: Birth Rate: Crude birth rate, indicating the number of live births per 1000 population during the year, estimated at midyear. Source: The World Bank; Dementia Incidence Rate: The number of new cases per 100,000 people diagnosed in 2019. Source: Institute for Health Metrics and Evaluation, University of Washington; Economic Affluence: Measured by per capita GDP (PPP), which is the per capita purchasing power parity value of all final goods and services produced within a territory in a given year (2018). Source: The World Bank; Biological State Index (I_bs_): Represents the level of genetic accumulation related to dementia due to reduced natural selection. Source: You & Henneberg, 2018; Life Expectancy at Birth (Aging): Source: The World Bank; Urbanization: Measured by the percentage of the population living in urban areas in 2018. Source: The World Bank.

In a stepwise linear regression model, aging emerged as the variable with the greatest impact on dementia incidence when birth rate was excluded. This indicates that, without considering birth rate, aging is the most significant predictor of dementia. However, when birth rate was included along with the other five independent variables, birth rate emerged as the most influential predictor of DIR with an *R*^2^ of 0.648. Economic affluence and I_bs_ followed, with the *R*^2^ increasing to 0.678 and 0.711 respectively. This suggests that economic affluence and urbanization do not account for a major part of the impact on DIR. These findings support our previous suggestion that birth rate is a significant predictor of DIR in the partial correlation analysis.

Table 4 displays the relationship between birth rate and DIR across different country groupings. The strength and significance of the correlation varied depending on the sample size and country characteristics. In the UN developed countries, the birth rate-DIR correlation was significantly stronger compared to UN developing countries (z = −2.97, *P* < 0.010 in Pearson’s r; z = −3.63, *P* < 0.001 in non-parametric). There was no significant difference in the birth rate-DIR correlation between the World Bank LMIC grouping and high-income grouping in the Pearson’s r model. However, a significant difference was observed in the non-parametric model (z = −2.01, *P* < 0.050).

**Table 4.** Birth Rate and Its Impact on the Incidence Rate of Dementia in Different Country Groupings.

Country groupings	Pearson r	*P*	nonparametric	*P*
Worldwide, n = 198	−0.797	<0.001	−0.910	<0.001
WHO regions				
African region, n = 47	−0.856	<0.001	−0.748	<0.001
Region of the Americas, n = 37	−0.792	<0.001	−0.828	<0.001
Eastern mediterranean region, n = 21	−0.488	<0.050	−0.369	0.100
European region, n = 53	−0.800	<0.001	−0.787	<0.001
South-east Asian region, n = 11	−0.783	<0.010	−0.818	<0.010
Western pacific region, n = 29	−0.672	<0.001	−0.834	<0.001
World bank income classifications				
High income countries, n = 47	−0.856	<0.001	−0.748	<0.001
Upper middle-income countries, n = 54	−0.803	<0.001	−0.847	<0.001
Low middle income countries, n = 29	−0.703	<0.001	−0.576	<0.001
Low-income countries, n = 49	−0.798	<0.001	−0.842	<0.001
Low- and middle-income countries (LMIC), n = 132	−0.824	<0.001	−0.921	<0.001
Fisher r-to-z transformation (LMIC vs high-income)	z = 0.62, *P* = 0.268		z = −2.01, *P* < 0.050	
United nations (common practice)				
Developed countries, n = 48	−0.466	<0.001	−0.518	<0.001
Developing countries, n = 150	−0.766	<0.001	−0.831	<0.001
Fisher r-to-z transformation (developing vs developed)	z = − 2.97, *P* < 0.010		z = − 3.63, *P* < 0.001	
Countries grouped with various factors				
Asia cooperation dialogue countries, n = 30	−0.618	<0.001	−0.651	<0.001
Asia-pacific economic cooperation countries, n = 20	−0.711	<0.001	−0.806	<0.001
Arab world countries, n = 21	−0.419	0.059	−0.262	0.251
European economic area, n = 29	−0.742	<0.001	−0.725	<0.001
English As official language countries, n = 56	−0.800	<0.001	−0.933	<0.001
Latin America countries, n = 24	−0.874	<0.001	−0.923	<0.001
Latin America and caribbean countries, n = 35	−0.766	<0.001	−0.798	<0.001
Organisation for economic Co-operation and development countries, n = 37	−0.782	<0.001	−0.855	<0.001
Southern African development community countries, n = 16	−0.854	<0.001	−0.874	<0.001
Shanghai Cooperation organization countries, n = 26	−0.650	<0.001	−0.664	<0.001

Data source & definition: Birth Rate: Crude birth rate, indicating the number of live births per 1000 population during the year, estimated at midyear. Source: The World Bank; Dementia Incidence Rate: The number of new cases per 100,000 people diagnosed in 2019. Source: Institute for Health Metrics and Evaluation, University of Washington.

## Discussion

This study highlights a significant inverse relationship between birth rate and dementia incidence rate (DIR). The findings indicate that countries with lower birth rates often have higher dementia rates. This trend persists even when adjusting for factors like aging, economic affluence, genetic predisposition, and urbanization.

Firstly, the study supports the previously established correlation between family size and dementia incidence rate.^15^ Lower birth rates in countries may result in higher rates of dementia. This finding supports the demographic transition hypothesis. It suggests that industrialization reduces birth rates, leading to an aging population and potentially higher dementia prevalence.^36,37^

Secondly, the analysis shows that birth rate independently predicts dementia incidence. The predictive power of birth rate remains strong even when factors like aging, economic affluence, genetic predisposition, and urbanization are considered. Notably, while birth rate is significant, economic affluence does not have the same impact when birth rate is accounted for. This indicates that birth rate plays a more crucial role in dementia risk than economic affluence.^38,39^

Thirdly, among the factors examined, birth rate has the most significant detrimental effect on dementia incidence rate, followed by genetic predisposition and aging. Economic affluence and urbanization have negligible influences on the development of dementia. This finding emphasizes the importance of focusing on demographic factors in dementia research and public health planning.^40,41^

Furthermore, the impact of low birth rate on dementia incidence is more pronounced in LMICs compared to high-income countries. Similarly, this effect is more significant in UN-designated developing countries than in developed countries.^1,42^ This difference may be attributed to variations in healthcare infrastructure, socioeconomic conditions, and cultural practices that influence family size and social support systems.^43^

The relationship between birth rate and dementia risk is influenced by parity and family size. Parity refers to the number of pregnancies a woman has carried to a viable gestational age. Higher average parity typically leads to a higher birth rate.^16,44,45^ Studies on parity and dementia risk have limitations and yield varying results depending on study design and population.^46^ However, larger family sizes, often associated with higher birth rates, have been linked to lower dementia risk due to increased social interactions and psychological support.^15^ The size of a family has a direct impact on the risk of developing dementia. In larger families, there are more opportunities for social interactions, which can contribute to improved psychological well-being and a lower risk of dementia.^47,48^ This connection between family relationships and dementia risk is supported by life course perspectives that consider biological, psychological, and social factors.^49,50^

In societies where there has been a shift from extended to nuclear families, there is a decrease in social interactions, potentially increasing the risk of dementia. This is especially notable in industrialized societies.^51^

Research has explored the therapeutic potential of oxytocin, a hormone associated with social bonding, in preventing and treating dementia. By reversing impairments caused by beta-amyloid in animal models, oxytocin shows promise in combating Alzheimer’s disease.^52,53^ Oxytocin also has positive effects on social and spatial memory, although the impact may vary depending on social contexts and individual attachment styles.^54,55^

Oxytocin offers therapeutic benefits beyond dementia treatment. It has been found effective for disorders related to social behavior, including autism spectrum disorder, posttraumatic stress disorder, schizophrenia, and anxiety disorders.^56,57^ This suggests that oxytocin could be useful in treating a range of neurological conditions.^58^

Vascular dementia, which is strongly linked to stroke, emphasizes the importance of preventing and managing cerebrovascular diseases.^59,60^ Oxytocin has been shown to have protective effects on the cardiovascular system, making it a potential treatment for vascular dementia.^61,62^

Frontotemporal dementia, which involves progressive damage to specific regions of the brain, also holds promise for oxytocin treatment. Empathy loss, a key symptom of frontotemporal dementia, may be mitigated by oxytocin’s ability to enhance empathy and prosocial behaviours.^63,64^ Studies have demonstrated improvements in behavioural symptoms with the use of intranasal oxytocin, indicating its therapeutic potential for frontotemporal dementia.^24,65,66^

Family dynamics are crucial in the prevention of dementia. Interpersonal interactions within families enhance psychological well-being. This improved well-being can provide biological protection against dementia.^49,67^ Positive psychosocial well-being, fostered by large families, can slow the development of dementia.^49^ Family support enhances psychological well-being and provides a sense of purpose in life, significantly reducing the risk of dementia.^67^

For pre-dementia patients, especially those with young onset dementia, a large family size is particularly important.^67^ Family members’ observations and encouragement can lead to early detection of atypical dementia symptoms. This, in turn, enables timely treatment and improves patient outcomes.^49^

From an evolutionary standpoint, populations with higher birth rates or larger family sizes may possess greater genetic diversity. This heightened diversity can be advantageous in terms of natural selection and potentially in reducing the risk of dementia.^68-74^ Large family sizes can reduce the persistence of dementia-causing genes within the population. This reduction can lead to a lower overall incidence of dementia .^75^

The correlation between birth rate and economic affluence, as well as dementia incidence, is significant. This supports the demographic transition theory.^76^ The correlation between birth rate and dementia incidence is particularly apparent in UN-developing countries and LMICs. In these regions, smaller family sizes and reduced social interactions contribute to a higher risk of dementia. UN-developing countries and LMICs often benefit from larger family sizes and informal healthcare support. This can help reduce the risk of dementia. These findings highlight the need for targeted public health interventions. They also underscore the importance of appropriately allocating resources to manage the growing burden of dementia in aging populations. When designing strategies for dementia prevention and care, policymakers and researchers should take into account demographic trends and family dynamics.

## Study Strength and Limitation

Little research has been conducted on the relationship between birth rate and dementia risk. This is likely due to the low occurrence of dementia, which makes it difficult to collect data. For example, the global average incidence rate of dementia is only 0.94%, which is not easily noticeable. To identify small birth rates as potential risk factors for dementia, a prohibitively large sample size would be needed for individual-based epidemiological or laboratory studies. However, ecological studies, which use aggregated quantitative data, can magnify the presence of dementia by 100,000 times, making it possible to analyze the effects of risk-modifying factors at the population level. Therefore, it is important to include ecological studies in the epidemiology of rare chronic diseases like dementia, cancer, and Type 1 diabetes.

However, it is important to note that this study has several intrinsic limitations due to its use of cross-sectional ecological data:

Firstly, each country is treated as a subject in this study, and the data used is aggregated. This means that the values for risk-modifying factors may not hold true for individuals who develop dementia.

Secondly, data that is aggregated or collected by international organizations may contain random errors due to the methods used. For example, the reported incidence of dementia depends on the reliability of diagnoses and may be affected by administrative errors. The quality of dementia incidence rate data also depends on the amount and quality of information available for each country. Generally, data from developing countries is less complete than data from developed countries.

Finally, there are over 100 types of dementia, but due to a lack of data, we are unable to explore the statistical role of birth rate in predicting each specific type of dementia.

## Conclusion

This study shows a strong inverse relationship between global birth rates and dementia incidence rates. The findings suggest dementia is a complex disease. Demographic shifts, like declining birth rates, are key predictors of higher dementia incidence. This holds true even when accounting for aging, economic affluence, genetic predispositions, and urbanization. Aging and genetic factors also influence dementia incidence. However, their impact seems less significant compared to birth rate trends.

## Data Availability

All the data used in this study are publicly and freely available from the official websites of the IHME and United Nations agencies. The aim of using these data in this study aligns with the terms and conditions set by the relevant international organizations. No formal permission is required to use the data for this study. The sources of the data have been described in the “Materials and Methods” section.*
